# T241. INTERPERSONAL COGNITIVE RIGIDITY AFFECTS SOCIAL FUNCTIONING IN PSYCHOSIS MORE THAN THEORY OF MIND: A STUDY WITH THE REPERTORY GRID TECHNIQUE

**DOI:** 10.1093/schbul/sby016.517

**Published:** 2018-04-01

**Authors:** Helena García-Mieres, Susana Ochoa, Victoria Furlan, Raquel Lopez Carrilero, Anna Villaplana, Regina Vila-Badia, Eva Grasa, Ana Barajas, Esther Pousa, Guillem Feixas

**Affiliations:** 1 Universitat de Barcelona; 2 Parc Sanitari Sant Joan de Déu; 3 Fundació Sant Joan de Déu; 4 IIB-Hospital Santa Creu I Sant Pau; 5 Centre de Salut Mental Les Corts; 6 Hospital del Mar

## Abstract

**Background:**

Social functioning impairment is one of the core features for schizophrenia diagnosis and are also present in other psychotic spectrum disorders, being determinant for disability. This impairment has multiple domains, which are linked but separate. Previous research has shown that social functioning is multiply determined by neurocognition, social cognition and symptoms, being social cognition the domain that accounts for more of the variance in daily functioning. However, cognitive rigidity in interpersonal perception has received less attention and much variance remains unexplained. The aim of this study was to test the role of interpersonal cognitive rigidity, as measured with the Repertory Grid Technique (RGT) in social functioning in psychosis.

**Methods:**

Sample of 40 out-patients with a psychotic spectrum diagnosis from the network of mental health services of Parc Sanitari Sant Joan de Déu (Barcelona, Spain). Cross-sectional study, assessment was carried out by a predoctoral researcher (GMH), using a sociodemographic questionnaire, the Social Functioning Scale (SFS), the Hinting Task (Theory of Mind, ToM), the Beck Cognitive Insight Scale (BCIS), and the RGT (to measure interpersonal cognitive rigidity, two indices were selected: Percentage of Variance Accounted for the First Factor, PVAFF, and Polarization). Pearson correlations and multiple regression analysis were performed.

**Results:**

Results showed that social engagement/withdrawal was explained by PVAFF, accounting for 16% of the variance. Independence-competence was explained by polarization, explaining 14.6% of the variance and by sex, which accounted for 11.1% of the variance. Independence-performance was explained by theory of mind, explaining 22.5% of variance. Employment/occupation was explained by years of illness accounting for 21.6% of variance, and by polarization (beta=-0.318, p=0.026) which explained 10% of variance. Finally, the total score of the SFS was explained by polarization, explaining 14.4% of variance, and sex, which accounted for 12.6% of variance. For prosocial activities and interpersonal communication, none of the variables entered for the linear regression analysis.

**Discussion:**

Despite ToM and cognitive insight are common variables reported in the research literature, in our study the cognitive rigidity measures of the RGT, based on the patients’ own terms (personal constructs) in rating their significant others, were better predictors of social functioning. These findings support the importance and utility of an idiographic instrument like the RGT to investigate cognitive processes related to social perception and their impact on functioning. Regarding PVAFF, a higher tendency to perceive the interpersonal world from a unidimensional manner predicted a worse outcome in social relationships/withdrawal. Regarding interpersonal polarized thinking, it was the best cognitive predictor of social functioning measures. Our results suggest that a dichotomous thinking style in interpersonal perception might also be relevant for elucidating the dysfunction in social adjustment domains. These findings are still preliminary, and form part of an ongoing study.

